# Persistent dyselectrolytemia in a neonate induced by liposomal amphotericin B. A case report

**DOI:** 10.3389/fped.2022.1099305

**Published:** 2023-01-10

**Authors:** Adrian Puertas Sanjuan, Carlos Javier Parramón-Teixidó, Susana Hernandez-Perez, Marie Antoinette Frick, Maria Jose Cabañas Poy

**Affiliations:** ^1^ Pharmacy Service, Vall D'Hebron Hospital Universitari, Barcelona, Spain; ^2^ Neonatology Service, Vall D'Hebron Hospital Universitari, Barcelona, Spain; ^3^ Infectious Pathology and Immunodeficiency Unit of Pediatrics, Vall d’Hebron Hospital Universitari, Barcelona, Spain

**Keywords:** liposomal amphotericin b [Ambisome(®)], tubulopathy, dyselectrolytemia, hypokalaemia, neonate

## Abstract

**Background:**

Nephrotoxicity is the most frequent serious adverse effect associated with amphotericin B deoxycholate treatment, for this reason, in recent years it has been relegated from routine clinical practice and replaced by the new liposomal formulations that have less nephrotoxicity. Nevertheless, dyselectrolytemia are a frequent adverse effect of the use of liposomal amphotericin B that usually are resolved with the withdrawal of the drug.

**Case presentation:**

We present a preterm neonate of 25 weeks gestation, with preserved renal function and most electrolytes within normal limits for gestational age except for mild hyponatremia in the first month of life. Due to an infection of the central nervous system and growth of Candida albicans, he required treatment with endovenous liposomal amphotericin B as well as intrathecal amphotericin B deoxycholate showing severe hydroelectrolyte disturbances and clinical worsening compatible with possible tubulopathy showing hypokalemia and severe hyponatremia a few days after starting treatment that persisted over time even after withdrawal of both drugs. Subsequently to the main alterations described, hypomagnesemia, hypophosphatemia, glycosuria and tubular proteinuria were also observed. Calcium levels remained stable after amphotericin B administration and did not require supplementation. In preterm or low birth weight newborns who present unjustified, severe and difficult to correct hydroelectrolyte disturbances despite the usual treatment, a possible tubulopathy should be considered, whether hereditary, primary or secondary to toxins or drugs.

**What Is New and Conclusion:**

We present the first case reported in a neonate in whom dyselectrolithemia has been maintained over time after withdrawal of liposomal amphotericin B.

## Background

Invasive fungal infection is a major cause of morbidity and mortality in immunocompromised patients. Amphotericin B has broad-spectrum antifungal activity and well-documented efficacy against *Candida spp., Aspergillus spp.* and *Cryptococcus spp.* infections (1).

Neonatal candidiasis is mainly diagnosed in the neonatal intensive care unit with described incidences of 18.8 per 1,000 live births under 1,000 grams. The main risk factor is prematurity, being extremely low-birth-weight infants (ELBW) with a weight <1,000 grams, the ones with the highest risk of invasive infection and as a complication, central nervous system (CNS) involvement. Antifungal treatment is established according to the sensitivity of the infecting species and the ability of the antifungal agent to reach adequate concentrations in blood or cerebrospinal fluid (CSF) (2).

According to the Infectious Diseases Society of America Guidelines, the first line of treatment for CNS fungal infection is amphotericin B deoxycholate (AMF-D) at a dose of 1 mg/kg daily. An alternative regimen is liposomal amphotericin B (AMF-L) at a dose of 5 mg/kg daily, which can be de-escalated to fluconazole at a dose of 6–12 mg/kg daily when the patient shows improvement on initial treatment. Treatment should be continued for at least 2 weeks until all signs, symptoms, CSF abnormalities have resolved and infected CNS devices have been removed (2).

Nephrotoxicity is the most frequent serious adverse effect associated with treatment with AMF-D, causing acute kidney injury in up to 50% of treated patients and tubular acidosis with loss of electrolytes in most patients. This is the reason why it has been relegated from clinical practice and replaced by AMF-L in both candidemia and CNS infections (2). Liposomal formulations stand out for their lower nephrotoxicity, however, they present hypokalemia as a frequent adverse effect of their use (1).

Usami et al. revealed that AMF-L can reduce plasma potassium concentrations if the renal tubules are damaged (1). The incidence of hypokalemia in patients treated with AMF-L was 36% in the study by Ringden et al. (3) and 51.3% in the work of Sunakawa et al. (4). In the study of Usami et al. conducted in adult patients, hypokalemia occurred in 53.8% of patients with recovery of concentrations 2–3 days after completion of AMF-L treatment (1).

On the other hand, in the study by Yamazaki et al. conducted in adult patients with hematologic disorders, approximately half of the hypokalemias improved after completion of AMF-L treatment, however, in some patients, it took more than 30 days to improve (5).

Possible tubulopathy should be considered, whether hereditary, primary or secondary to toxins or drugs in those preterm or low birth weight newborns who present unjustified, severe and difficult to correct hydroelectrolytic alterations in spite of the usual treatment (6).

## Case presentation

Preterm new-born male, 25 weeks gestation age with a birth weight of 680 grams, presenting placental abruption, loss of fetal well-being and severe hyaline membrane disease, requiring surfactant administration, high frequency mechanical ventilation and vasoactive drugs after birth. The main risk factor for placental abruption was premature rupture of membranes. At 2–3 days of life he presented intraventricular haemorrhage, which rapidly progressed to dilatation, requiring external ventricular drain (EVD) at 10 days of life.

During the first month of life, presented mild hyponatremia with sodium values of around 132 mmol/L in venous blood, which were rapidly corrected with intravenous sodium reposition at 6 mEq/kg/day. It should be noted that both renal function and other electrolytes remain within normal limits for her gestational age.

At 29 days of life, and with a previous history of nosocomial catheter-associated sepsis due to *Staphylococcus haemolyticus* and treated for 15 days with vancomycin, the CSF culture extracted from the EVD showed growth of *Candida albicans*, which was confirmed in a second culture 3 days later. Blood culture was performed with negative results. At 32 days of life it was decided to start AMF-L at 5 mg/kg and subsequently withdraw the EVD.

**Figure 1:**
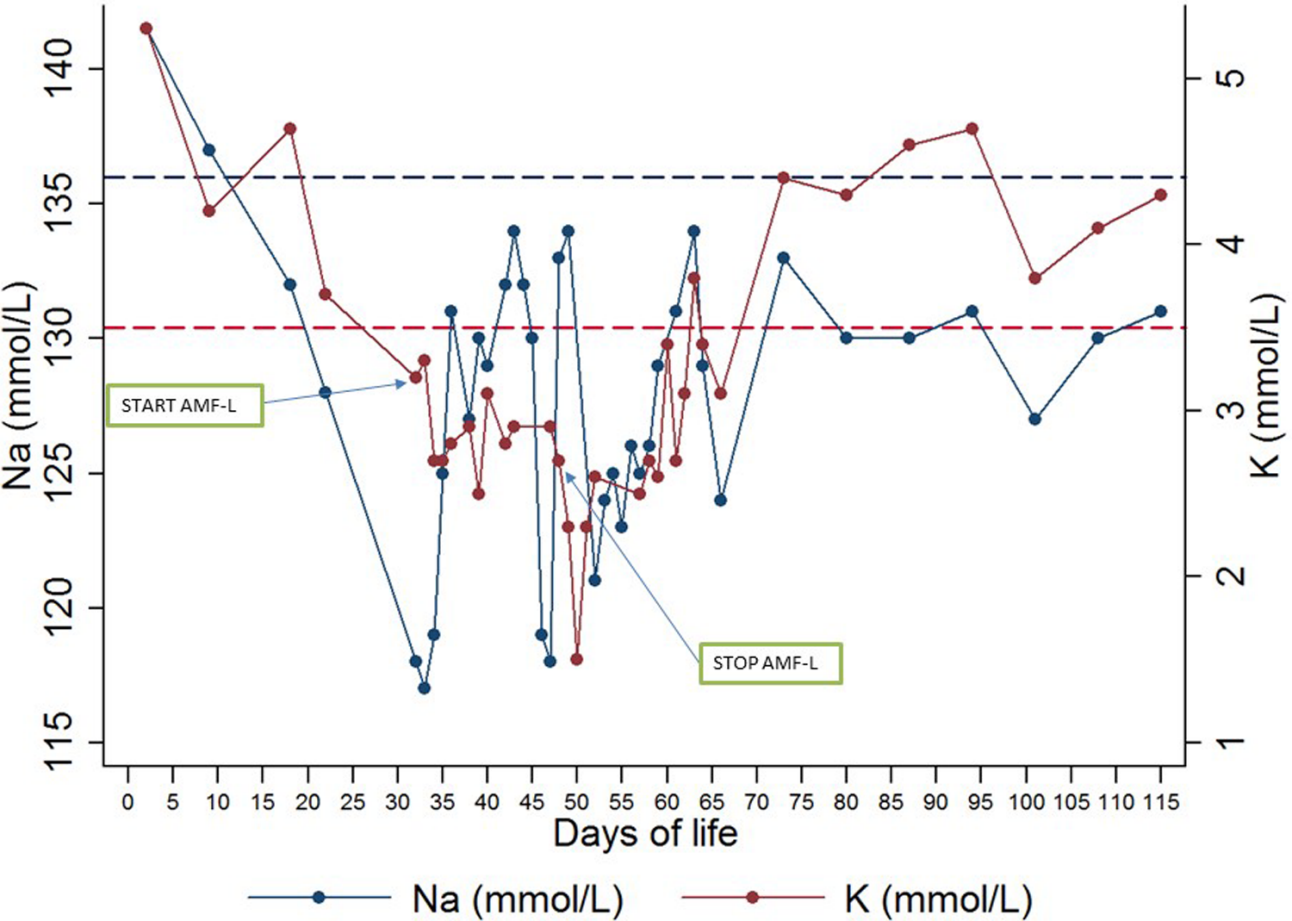
Evolution of sodium and potassium over time. Blue dash line: lower limit of hyponatremia. Red dash line: lower limit of hypokalemia.

Two days after starting treatment with AMF-L, develops hyponatremia with sodium values of 117–119 mmol/L requiring intravenous correction to 9 mEq/kg/day (Figure 1).

At 36 days of life, he presented clinical worsening compatible with possible tubulopathy with hypokalemia (2.8 mmol/L (reference value (RV): 3.5–5.1)), hypomagnesemia (1.8 mg/dl (RV: 1.8–2.6)) and hyponatremia (131 mmol/L (RV: 136–146)) (Figure 1). In this context, an electrolyte replacement was performed with 5% glucose saline at 100 ml/kg/day and with sodium (5 mEq/kg/day), potassium (2 mEq/kg/day) and magnesium (0.3 mEq/kg/day).

At 40 days of life, treatment with intrathecal AMF-D (25 mcg every 72 h) was started due to persistence of yeasts in CSF after placement of the new EVD. Subsequently, at 48 days of life, AMF-L and intrathecal AMF-D were discontinued (after 16 and 7 days of treatment, respectively) and treatment was de-escalated to fluconazole due to good clinical evolution and negative result of control cultures of CSF.

These dyselectrolytemia are maintained for days showing potassium concentrations of 2.5 and 3.1 mmol/L at 39 and 40 days of life, respectively. At 53 days of life a urine ion study was performed showing renal ion losses and proteinuria. Hypophosphatemia was also observed with levels of 3.0 mg/dl (RV: 4.8–7.2 mg/dl). At 60 days of life the patient shows a tendency to be more edematous with worsening hyponatremia of 124 mmol/L, hypokalemia of 2.4 mmol/L and hypoosmolar hyponatremia is diagnosed. It should be noted that potassium levels remained low until 66 days of life with values of 3.1 mmol/L (Figure 1). In view of this situation, the Pediatric Nephrology Service was consulted and the diagnosis was suggested as a possible proximal tubulopathy with proteinuria in the mixed nephrotic range and tubular predominance, possibly induced by drugs.

**Figure 2:**
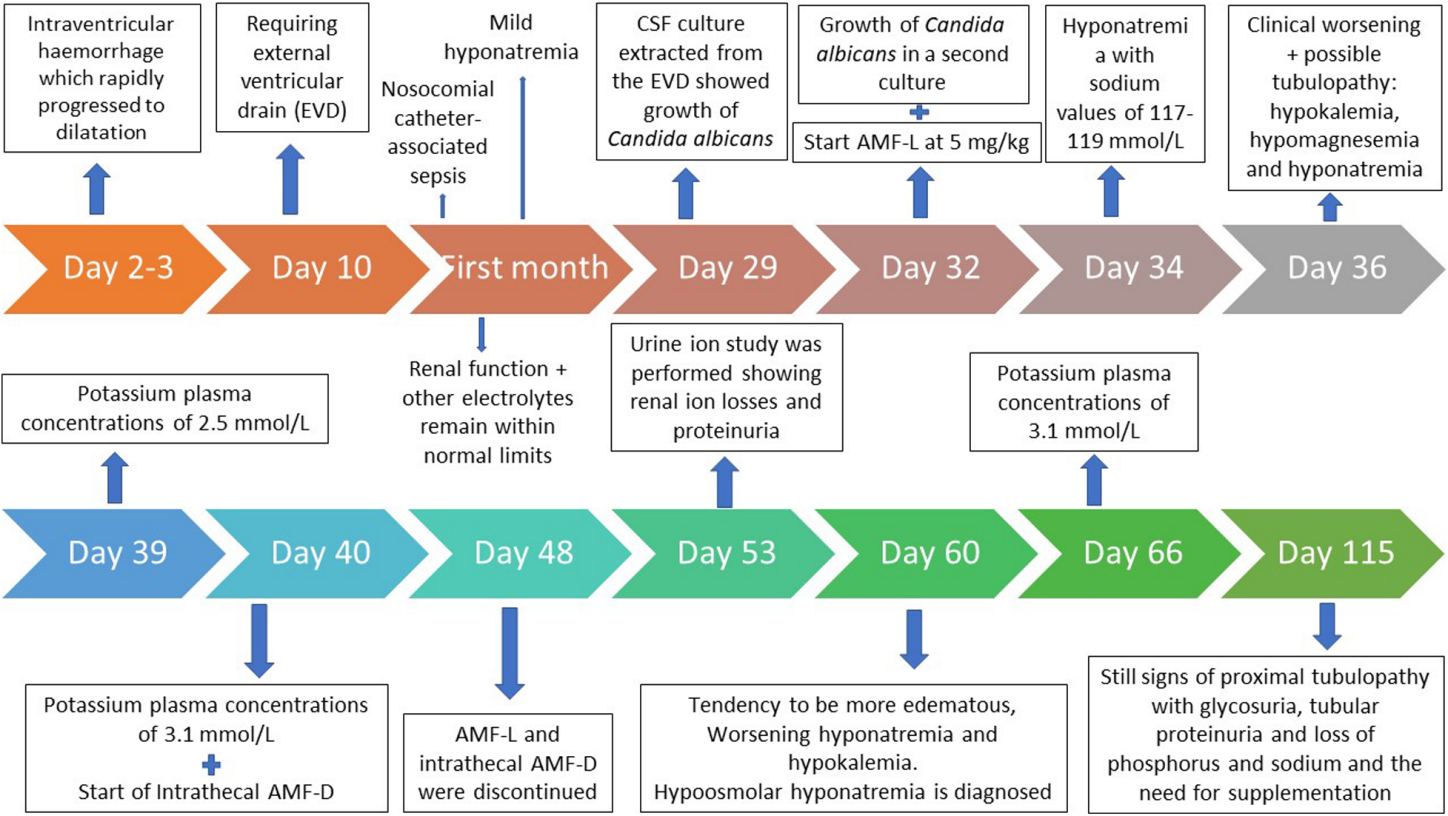
Investigations date wise and flowchart of patient evolution.

Finally, at 115 days of life, there were still signs of proximal tubulopathy with glycosuria, tubular proteinuria and loss of phosphorus and sodium and the need for supplementation (oral sodium at 5 mEq/kg/day and phosphate at 2 mEq/kg/day) (Figure 2). The patient did not show hypercalciuria and calcium levels remained stable after amphotericin B administration and did not require supplementation.

For clinical follow-up, a complete urine study was performed every 2 weeks, together with a blood biochemical analysis and gasometry. Necessary hydroelectrolytic restoration was continued in order to maintain the hydroelectrolytic balance as stable as possible.

Complementary tests were performed to exclude possible underlying pathologies: Ultrasound showed kidneys with normal structure and nephrocalcinosis was ruled out. Possible congenital causes related to proximal tubule dysfunction and hydroelectrolyte alterations were evaluated. It is worth mentioning that glomerular function was preserved at all times.

## Discussion and conclusion

We present the first case reported of a neonate in whom dyselectrolytemia persisted over time (Figure 1), even after withdrawal of the suspected drug (AMF-L). In the study of Usami et al. performed in adult patients the dyselectrolytemia resolved 2–3 days after withdrawal of the drug involved, whereas in the study by Yamazaki et al. hypokalemia tended to persist in the long term after termination of AMF-L (1, 5). Dyselectrolytemia was classified as possibly related to AMF-L administration according to Naranjo's causality algorithm (7). We should bear in mind that the patient profile of this last study is completely different from the one we present.

Tubulopathies comprise a heterogeneous group of entities that lead to profound alterations in electrolyte homeostasis. Hereditary or primary tubulopathies must be distinguished from those secondary to toxins, drugs or other diseases and it is important to classify them according to the area of the tubule affected (8, 9).

Fanconi syndrome can be primary or secondary (exposure to certain drugs, associated with acquired diseases or genetic diseases), and is characterised by proximal tubule failure leading to hyperchloremic metabolic acidosis, hypokalemia, glycosuria, hypophosphatemia, aminoaciduria, tubular proteinuria and vitamin D-resistant rickets. In the presence of clinical alterations that may be compatible with this syndrome, as in the case of our patient, secondary forms should always be ruled out first. The evolution of this syndrome is usually variable, depending on the etiologic agent; some cases disappear when the causal agent is suppressed or the underlying disease is treated. In the primary forms, the clinical course is usually chronic and the evolution is progressive towards chronic disease (8, 9).

In this case, differential diagnosis should be made with other causes of congenital proximal complex tubulopathies, such as cystinosis and galactosemia (8, 9). Cystinosis represents the most frequent cause of Fanconi syndrome of genetic etiology and in infants should be considered first in the differential diagnosis (10). Other etiologies should be highlighted in the presence of Fanconi syndrome, such as tyrosinemia, galactosemia, fructose intolerance, Wilson's disease, Lowe's syndrome, glycogenosis, heavy metal intoxication, glycoglycinuria and others (9).

Cystinosis was ruled out after ophthalmologic evaluation, absence of hepatomegaly, absence of nephrocalcinosis in renal ultrasound and because it has a later presentation (10, 11).

Galactosemia is a group of genetic disorders of galactose metabolism that induce a series of variable clinical manifestations and was ruled out with neonatal screening (12).

Regarding hyponatremia and edema, it could be related to SIADH in the context of cerebral pathology and which worsened at the time due to fluid overload (13).

Extreme prematurity implies tubular immaturity, but in the clinical context of this patient, tubulopathy could be of pharmacological cause. In this case, prematurity probably contributed to the fact that the dyselectrolytemia was maintained long after AMF-L was discontinued (6).

Knowing the adverse drug effect profile is critical to achieving treatment success, especially in patient populations, such as preterm infants, for whom information is scarce if not nonexistent. The integration of the pharmacist in the multidisciplinary team is a strategy to detect adverse reactions and advance patient safety.

## Data Availability

The raw data supporting the conclusions of this article will be made available by the authors, without undue reservation.
